# Maresin 1 protects the liver against ischemia/reperfusion injury via the ALXR/Akt signaling pathway

**DOI:** 10.1186/s10020-021-00280-9

**Published:** 2021-02-25

**Authors:** Da Tang, Guang Fu, Wenbo Li, Ping Sun, Patricia A. Loughran, Meihong Deng, Melanie J. Scott, Timothy R. Billiar

**Affiliations:** 1 Department of General Surgery, The Third Xiangya Hospital, Central South University, 410000 Changsha, People’s Republic of China; 2 Department of Burn and Plastic Surgery, The Second Xiangya Hospital, Central South University, 410000 Changsha, People’s Republic of China; 3 Department of Hepatobiliary Surgery, Union Hospital, Huazhong University of Science and Technology, Wuhan, People’s Republic of China; 4 Department of Surgery, Ohio State University Medical School, OH Columbus, USA; 5 Department of Surgery, University of Pittsburgh, PA 15213 Pittsburgh, USA; 6 Pittsburgh Trauma Research Center, University of Pittsburgh, 15213 Pittsburgh, PA USA; 7 Pittsburgh Liver Research Center, University of Pittsburgh, 15213 Pittsburgh, PA USA

**Keywords:** Lipid mediators, Hepatic ischemia/reperfusion, Hepatocytes, Inflammation, Oxidative stress, Apoptosis

## Abstract

**Background:**

Hepatic ischemia/reperfusion (I/R) injury can be a major complication following liver surgery contributing to post-operative liver dysfunction. Maresin 1 (MaR1), a pro-resolving lipid mediator, has been shown to suppress I/R injury. However, the mechanisms that account for the protective effects of MaR1 in I/R injury remain unknown.

**Methods:**

WT (C57BL/6J) mice were subjected to partial hepatic warm ischemia for 60mins followed by reperfusion. Mice were treated with MaR1 (5-20 ng/mouse), Boc2 (Lipoxin A4 receptor antagonist), LY294002 (Akt inhibitor) or corresponding controls just prior to liver I/R or at the beginning of reperfusion. Blood and liver samples were collected at 6 h post-reperfusion. Serum aminotransferase, histopathologic changes, inflammatory cytokines, and oxidative stress were analyzed to evaluate liver injury. Signaling pathways were also investigated in vitro using primary mouse hepatocyte (HC) cultures to identify underlying mechanisms for MaR1 in liver I/R injury.

**Results:**

MaR1 treatment significantly reduced ALT and AST levels, diminished necrotic areas, suppressed inflammatory responses, attenuated oxidative stress and decreased hepatocyte apoptosis in liver after I/R. Akt signaling was significantly increased in the MaR1-treated liver I/R group compared with controls. The protective effect of MaR1 was abrogated by pretreatment with Boc2, which together with MaR1-induced Akt activation. MaR1-mediated liver protection was reversed by inhibition of Akt.

**Conclusions:**

MaR1 protects the liver against hepatic I/R injury via an ALXR/Akt signaling pathway. MaR1 may represent a novel therapeutic agent to mitigate the detrimental effects of I/R-induced liver injury.

## Background

Liver ischemia/reperfusion (I/R) injury is a crucial contributor to liver damage and dysfunction after liver transplantation, partial hepatectomy, and hemorrhagic shock (Chen et al. 2020). Although great efforts have been made to explore therapeutic strategies to alleviate acute liver I/R injury, no pharmacologic intervention has been documented to be effective in preventing or treating this condition in clinical practice (van Golen et al. 2013). Therefore, more exploration into the potential mechanism and preventive strategies for hepatic I/R injury is urgently needed.

The pathophysiological process of liver I/R injury includes cell damage directly induced by ischemia, and subsequent severe hepatocyte damage caused by reperfusion-related inflammation (Guo et al. 2020, Zhang et al. 2018). During ischemia, hepatocytes are subjected to metabolic disturbance, which can directly initiate cell death. During reperfusion, the generation of reactive oxygen species (ROS) disturbs cellular redox status, which further contributes to cellular injury (Malhi et al. 2008). In response to oxidative stress, the recruitment and activation of Kupffer cells (KCs), monocytes and neutrophils produce damage-associated immune responses that can lead to hepatocyte apoptosis (Lentsch et al. 2000). Based on these observations, targeting the inflammatory response, apoptosis, and oxidative stress are promising approaches for ameliorating I/R related liver injury.

The n-3 polyunsaturated fatty acids (PUFAs), including eicosapentaenoic acid (EPA), arachidonic acid (AA), and docosahexaenoic acid (DHA), are dietary components and play important roles in many physiological processes (Chiang et al. 2019). A recent study emphasizes that endogenous n-3 PUFA-derived specialized pro-resolving mediators (SPMs), including maresins, lipoxins, protectins and resolvins, have potential anti-oxidative and anti-inflammatory properties (Serhan 2014). Maresins are biosynthetic derivatives of DHA synthesized in macrophages (Serhan et al. 2009). Recently, a number of studies have revealed that MaR1 exerts powerful anti-inflammatory and pro-resolving effects in several disease models by promoting the resolution of inflammation through reduced neutrophil infiltration and improved macrophage phagocytic activity without causing immunosuppression (Han et al. 2019, Gong et al. 2015, Buckley et al. 2014). Furthermore, studies also show that MaR1 has protective effects on I/R injury in several organs including the liver in a rat model (Soto et al. 2020). However, the mechanisms of the protective effects of MaR1 in hepatic I/R injury have not been established.

The aim of the current study was to examine the effect of MaR1 on hepatic damage in a mouse model of liver I/R and to define the mechanisms by which MaR1 reduces hepatocellular death during hepatic I/R injury. Our investigations highlight an unrecognized role for the ALXR (lipoxin A4 receptor) /Akt signaling pathway in MaR1-mediated antioxidant defenses during hepatic I/R injury.

## Materials and methods

### Animal

Male C57BL/6J wild-type (WT) were purchased from Jackson Laboratory and were bred at our animal facility. All experimental mice weighing 25–28 g used in this study were male and 8–12 weeks old. Animal protocols were approved by the Animal Care and Use Committee of the University of Pittsburgh and the experiments were performed in strict adherence to the NIH Guidelines for the Use of Laboratory Animals.

### Reagents

Western blot antibodies were purchased from Cell Signaling Technology and Novus Biologicals; LY294002 was obtained from Sigma-Aldrich. Mouse cytokine ELISA Kits were purchased from R&D Systems. MaR1, MDA assay kit, MaR1 Elisa kit, and GPX activity assay kit were purchased from Cayman Chemical. Boc2 was obtained from Phoenix Pharmaceuticals, Inc. Lactate dehydrogenase (LDH) kit was purchased from Thermo Scientific. CCK-8 kit was obtained from Dojindo Molecular Technologies, Inc.

### Liver I/R model

A nonlethal segmental (70 %) hepatic warm ischemia-reperfusion model was used as previously described (Tsung et al. 2005). Briefly, the blood supply of the left and median liver lobes was interrupted with a microvascular clamp for 1 h and reperfusion was initiated by removing clamps. The temperature of the mice during the period of ischemia was maintained at 33 °C using an incubator chamber. Sham-operated mice underwent the same surgical procedure without vasculature occlusion. Serum and liver samples of mice were collected 6 h after reperfusion. MaR1 (5-20 ng/mouse; diluted with PBS) or PBS (control) was injected via tail vein at the beginning of reperfusion. Boc2 (50 mg/kg) or vehicle control was administrated into peritoneum 1 h before the surgical procedures. LY294002 (0.5 mg/kg, i.p.) was injected 30 min before I/R surgery.

### Isolation, culture, and treatment of hepatocytes and nonparenchymal cells

Hepatocytes (HCs) were isolated from mice as previously described (Lei et al. 2018). Briefly, HCs were separated from nonparenchymal cells (NPCs) through two cycles of differential centrifugation and further purified with a 30 % Percoll gradient. HC purity exceeded 99 % and HC cell viability was more than 95 %. NPCs were isolated and purified without HCs as described (Yi et al. 2020). For HC&NPC co-culture, freshly isolated mouse HCs were seeded in collagen-coated 24-well plates at 1.5$\times$10^5^ cells/well in Williams’ E medium and incubated for 2-4 h. Then, HCs were washed with PBS and overlaid with NPCs (7.5$\times$10^5^ cells/well) and incubated together. For experiments involving hypoxia, the cells were placed into a modular incubator chamber, which was equilibrated with the anoxic gas mixture (94 % N_2_, 5 % CO_2_, and 1 % O_2_.). For experiments using MaR1, 10 nM, 30 nM or 100 nM was added to the cell media 30mins prior to treatment with hypoxia. After incubating under hypoxia for 10 h, primary cells were incubated under normoxic conditions (air/5 % CO2) for another 10 h. The cells and supernatants were collected for further analysis.

### Liver damage assessment

To assess the cellular injury and hepatic function following liver I/R injury, serum alanine aminotransferase (sALT) and serum aspartate transaminase (sAST) were evaluated using DRI-CHEM 4000 Chemistry Analyzer System (Heska). The percent necrotic area in the ischemic lobes was determined by the random assessment of each H&E stained histological section using Image J software. Liver sections were scored using Suzuki methodology for characterizing I/R induced liver damage (Suzuki et al. 1993).

### Elisa analysis

Serum IL-6, IL-10 and IL-1β levels in the mice were detected by ELISA kits (R&D Systems) according to the manufacturer’s instructions. Serum Maresin 1 levels in the mice subjected to sham operation and I/R surgery were measured by Elisa kit (# 501,150, Cayman chemical) following the manufacturer’s protocols.

### Measurement of malondialdehyde (MDA) and Glutathione peroxidase (GPX) levels

Frozen liver samples were weighed and homogenized. After centrifugation, the supernatants were collected for further experiments. MDA level and GPX activity were estimated by their respective commercial assay kits (Cayman Chemical) according to the manufacturer’s protocol.

### Immunofluorescent staining

Liver samples were prepared as described. Briefly, ischemic liver lobes were cryoprotected in 2 % paraformaldehyde for 2 h and then 30 % sucrose for another 24 h. After that, the liver samples were cut into 6${\upmu }\text{m}$ sections and placed onto slides. Immunofluorescent staining was begun by rehydrating the slides with PBS. Then, the liver sections were blocked with PBS + 20 % normal goat serum (NGS) for 45mins. All samples were incubated with the specific primary antibody for lymphocyte antigen 6G [Ly6G] (1:100, BD Biosciences, catalog 560,599) for 60 mins. The sections were washed with 0.5 % BSA three times and then the secondary antibody was incubated for another 60mins. Finally, all groups were stained for F-actin and nuclear staining. Cell death was evaluated by incubating with In Situ Cell Death Detection TMR Red (1:1000 Roche no. 12,156,792,910) according to the manufacturer’s protocol. Images were taken by a Nikon A1 confocal microscope. Quantification was performed using NIS Elements (Nikon).

### Immunoblotting

Western blot analysis was performed using whole-cell lysates from either ischemic liver lobes or HCs as previously described (Huang et al. 2014). The membranes were blocked in 5 % milk for 1-2 h and then incubated overnight using the following primary antibodies (Cell Signaling Technology): β-actin, ERK, phospho-ERK, JNK, phospho-JNK, p38, phospho-p38, Akt, phospho-Akt, Bcl-2-associated X protein (Bax), B-cell lymphoma 2 (Bcl-2), cleaved-caspase3, and ALXR (Novus Biologicals). Membranes were washed in TBST for 10mins, incubated with HRP-conjugated secondary antibody for 60mins, and then washed for 30 min in TBST before being detected by chemiluminescence (Thermo Fisher Scientific).

### Real-time polymerase chain reaction (PCR) analysis

High quality total RNA was extracted from frozen liver tissues with the RNeasy Mini Kit (Qiagen) according to the manufacturer’s protocols. Complementary DNA (cDNA) was generated from 1ug RNA by Reverse Transcription Supermix (Bio-Rad #1,708,841). cDNA was then assayed by real-time PCR (RT-PCR) in duplicate using specific primers (Primer sets: ALXR, QT00171514, Qiagen; LGR6, QT00296632, Qiagen) and universal SYBR Green Supermix (Bio-Rad #1,725,121). GAPDH was used as an endogenous control.

### Lactate dehydrogenase assay

Lytic cell death in HCs subjected to H/R treatment was evaluated by measurement of lactate dehydrogenase (LDH) released into the cell medium. After centrifugation, 50μL of the medium was transferred to a 96-well plate, and the levels of LDH were analyzed using Pierce LDH Cytotoxicity Assay Kit (Thermo Scientific) according to the manufacturer’s instructions.

### Cell viability assay

To evaluate the effects of MaR1 on cell viability, primary HCs (2 × 10^4^ cells/mL) and NPCs (1 × 10^5^ cells/mL) were seeded into 96-well plates in a volume of 100uL per well. After treatment with MaR1 for 24 h, the CCK-8 kit was used to measure the cell viability.

### Statistics

Data analysis was performed using GraphPad Prism software. Results are shown as the mean$\pm$SEM. Comparisons between two experimental groups were performed by Student’s t test. One-way analysis of variance (ANOVA) followed by post hoc Tukey test was used for multiple comparisons (more than two groups). Differences were considered significant at *P*$<$0.05.

## Results

### Maresin1 alleviates liver I/R injury in a dose‐dependent manner

**Fig. 1:**
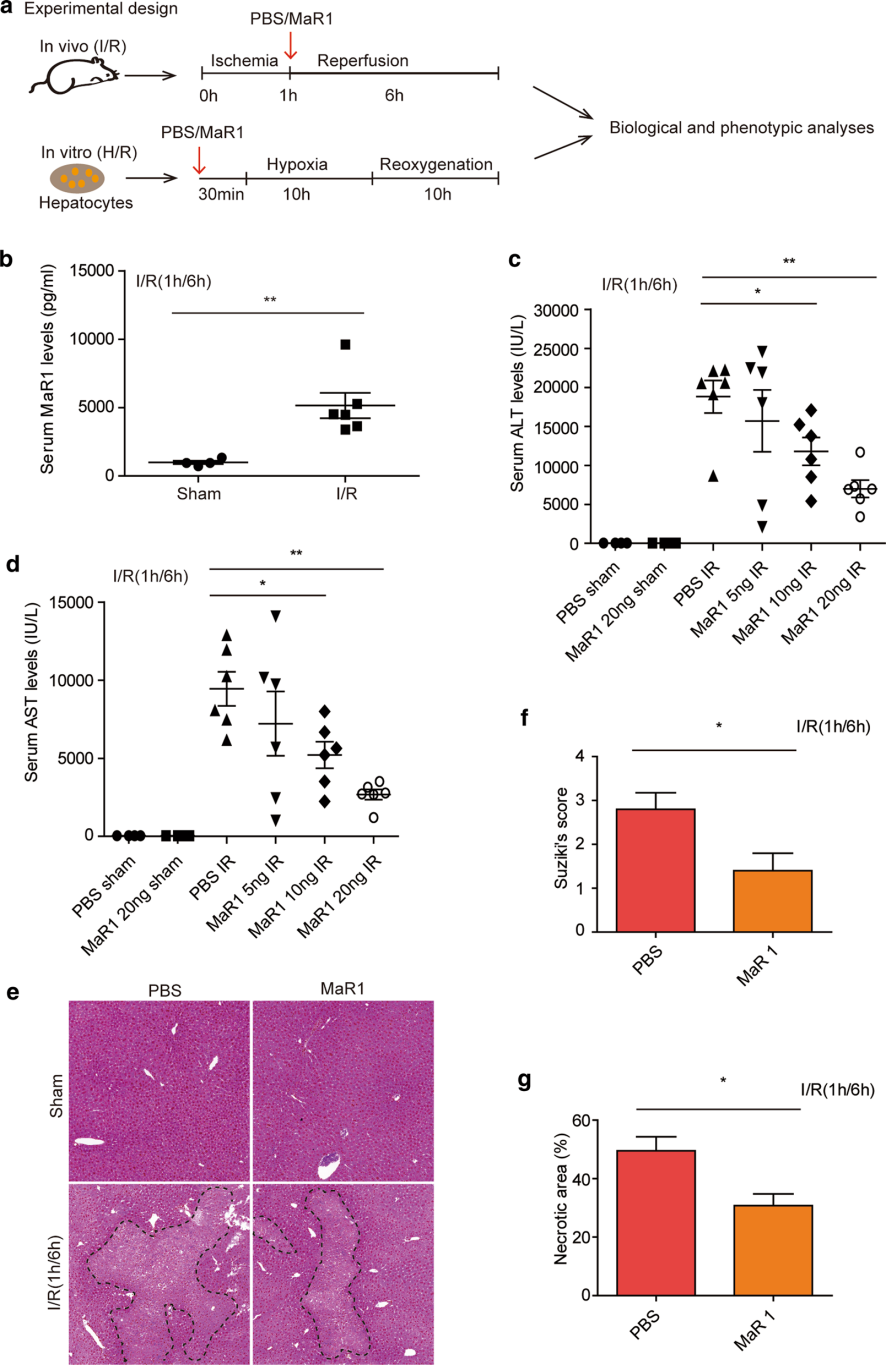
Low-dose MaR1 prevents liver I/R injury. WT mice were injected via tail vein with either PBS (control) or MaR1 at doses of 5 ng, 10 ng or 20 ng/mouse at the beginning of reperfusion after 1 h ischemia. **a** Graphical scheme of the experimental design. **b** Serum MaR1 levels of WT mice subjected to hepatic I/R for 6 h or a sham procedure (n = 4 for sham group; n = 6 for I/R group). Serum ALT (**c**) and AST (**d**) at 6 h post-reperfusion. Plots show levels in individual mice (n = 6/gp) with bars showing mean ± SEM. Liver H&E (original magnification ×20) from WT mice given PBS (control) or MaR1 (20 ng/mouse) after 1 h ischemia followed by 6 h reperfusion or sham procedure (**e**). (**f**) Suzuki’s histological score of liver damage (n = 5/gp); **g** Dotted lines indicate measured necrotic areas (quantified in bar graph (n = 5/gp); Images are representative across all measured samples. Data are presented as mean ± SEM. *P$<$0.05, **P$<$0.01

A graphical representation of the experimental approach is shown in (Fig. 1a). To address whether MaR1 is associated with liver I/R injury, we first assessed serum MaR1 levels in WT mice subjected to sham operation or partial hepatic ischemia followed by 6 h of reperfusion. Elisa analysis showed that serum MaR1 was at low levels in the sham group but was significantly increased to around 5 ng/ml following hepatic I/R injury (Fig. 1b). Next, to determine whether MaR1 could prevent liver I/R injury in mice, WT mice were given i.v. injections of PBS or different doses of MaR1 at the beginning of reperfusion. Liver injury was examined at 6 h post-reperfusion. Treatment with 5 ng/mouse MaR1 had no effect on serum ALT (sALT) and serum AST (sAST) levels, however, doses of 10 ng/mouse and 20 ng/mouse MaR1 significantly reduced I/R-induced sALT and sAST levels (Fig. 1c, d), showing that MaR1-mediated protection from I/ R induced liver injury was dose-dependent. The optimal effect of MaR1 was observed at 20 ng/mouse. Hence, 20 ng/mouse MaR1 was chosen for our subsequent experiments. Severe hepatocellular necrosis was present in liver sections from mice that were treated with PBS, whereas necrotic areas were significantly reduced in liver samples from MaR1-treated mice (20 ng/mouse) (Fig. 1e). The extent of necrosis and Suzuki’s histological scores confirmed by H&E staining of liver tissues were consistent with sALT and sAST levels (Fig. 1f, g). Together, these data indicate that MaR1 can be induced following I/R mediated liver damage, and the administration of MaR1 confers protection against hepatic I/R injury in a mouse model.

### MaR1 protects hepatocytes from cell death both in vivo and in vitro

**Fig. 2:**
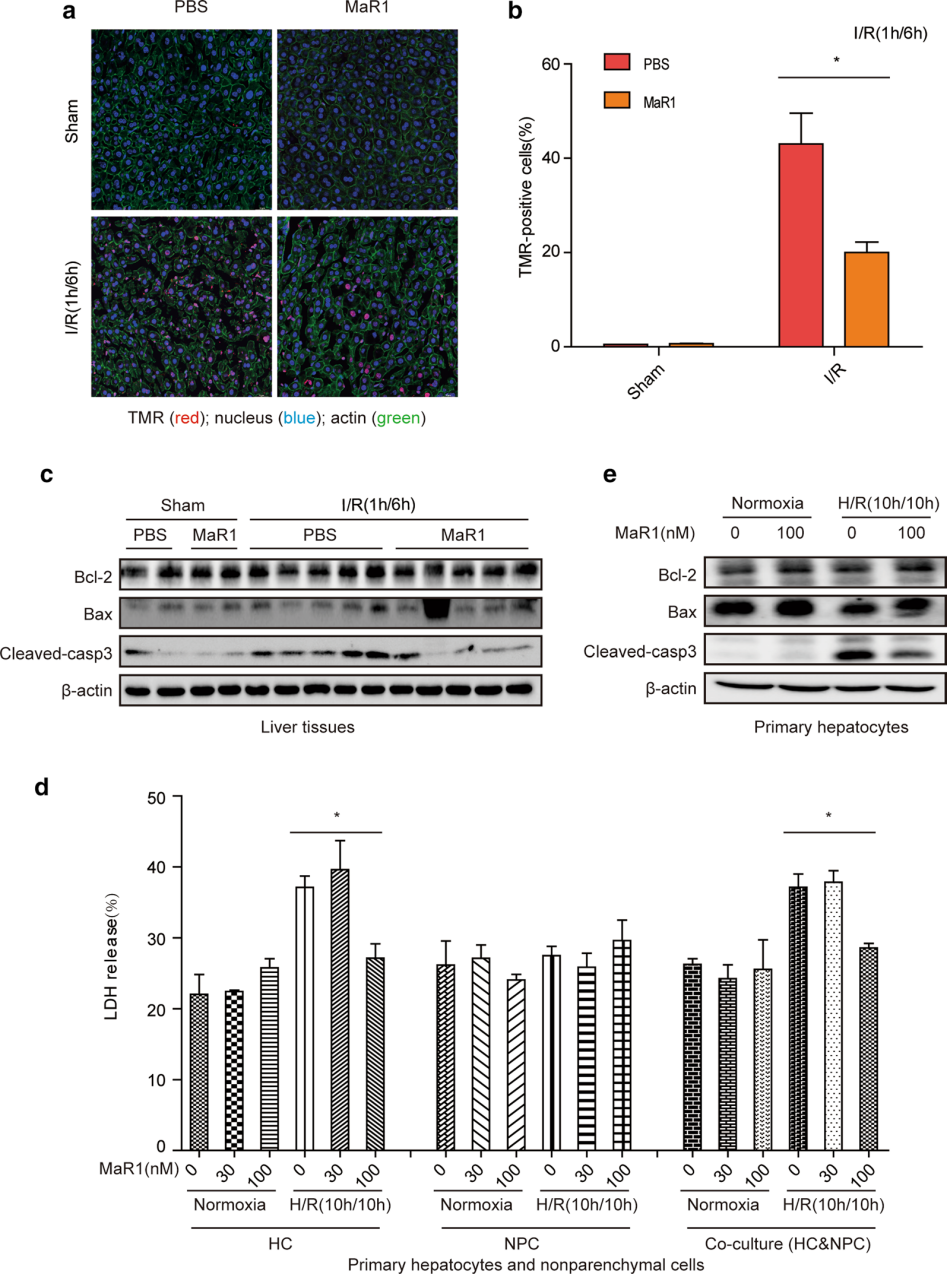
MaR1 reduces hepatocyte apoptosis during hepatic I/R injury. **a** Confocal images of TMR (red), nucleus (blue), and F-actin (green) in liver sections obtained from PBS or MaR1-treated mice subjected to sham surgery or 1 h ischemia/6 h reperfusion (scale bar = 25 μm). **b** Percentage of TMR-positive cells per liver section (n = 3 or 4/gp). **c** Western blot analysis of apoptosis markers (Bax, Bcl-2 and cleaved-caspase3) in liver tissues from PBS- and MaR1-treated mice that underwent sham operation or liver I/R insult (1 h/6 h). **d** LDH release in primary mouse HCs, NPCs, or HC/NPC co-cultures subjected to normoxia or 10 h hypoxia (1% O2)/10 h reoxia (H/R 10 h/10 h) without (0) or with pretreatment with 30 or 100 nM MaR1. **e** Western blot analysis of Bcl-2, Bax and cleaved-caspase3 in primary mouse HCs treated with normoxia or H/R without (0) or with 100 nM MaR1 given prior to H/R. Images representative of at least 3 separate experiments. All data are shown as mean ± SEM. *P$<$0.05

Hepatocellular apoptosis is prominent in hepatic I/R injury (Malhi et al. 2006). To further investigate the protective role of MaR1 in the liver following I/R insult, TMR staining, a method to detect single-and double-stranded DNA breaks, was employed to assess the extent of cell death during hepatic I/R injury (in vivo). There were fewer TMR-positive cells in the livers of the MaR1-treated I/R group than in those of PBS-treated mice (Fig. 2a, b). In addition, several pro-apoptotic and anti-apoptotic factors in ischemic liver lobes were evaluated by Western blotting. As expected, lower cleaved-caspase 3 protein levels were observed in livers from MaR1-treated mice compared with PBS-treated mice (Fig. 2c), indicating MaR1 protected liver cells from apoptosis after I/R. However, Bcl-2 and Bax levels were comparable between the two groups (Fig. 2c). To further assess the role of MaR1 in vitro, cultured primary hepatocytes (HCs) and nonparenchymal cells (NPCs) were treated with increasing concentrations of MaR1, and a CCK-8 assay was used to evaluate the cell viability. MaR1 concentrations of less than 300 nM did not affect cell viability under normoxic conditions (Additional file 1: Fig. S1). Primary HCs and NPCs were isolated from WT mice and subjected to hypoxia (1 % O_2_) and reoxygenation (H/R; 10 h hypoxia/10 h reoxygenation) in the presence or absence of MaR1. Lytic cell death was assessed by LDH release into the medium. MaR1 (100 nM) dramatically suppressed LDH release in primary HCs cultured alone or with NPCs, whereas the protective effects of MaR1 were not observed in NPCs cultured alone (Fig. 2d). In line with the in vivo results, MaR1 also resulted in lower levels of hepatocyte apoptosis when compared with vehicle-treated hepatocytes after H/R, as demonstrated by lower cleaved-caspase 3 protein levels (Fig. 2e). Collectively, these findings suggest that MaR1 is capable of reducing oxidative stress-induced cell death in HCs independent of NPCs.

### MaR1 suppresses inflammatory responses following liver I/R injury

**Fig. 3:**
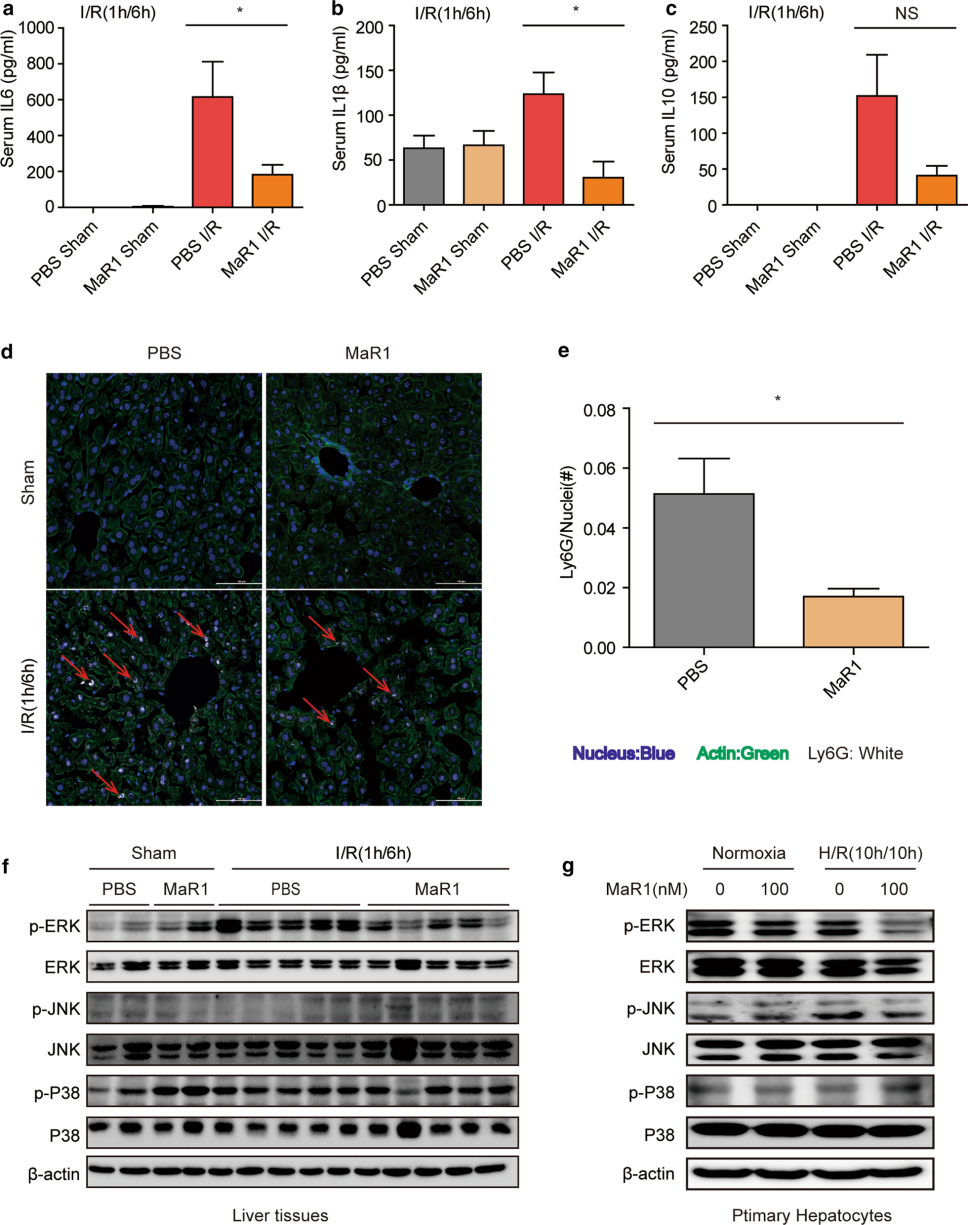
MaR1 inhibits inflammatory responses following hepatic I/R injury. Serum levels of cytokines IL-6 (**a**), IL-1β (**b**) and IL-10 (**c**) in WT mice after sham or I/R (1 h/6 h) treated with PBS or MaR1 (20 ng/mouse) (n = 6/gp). **d** Representative immunofluorescence staining of LY6G-positive inflammatory cells in ischemic lobes of mice in the indicated groups. Ly6G (white), nucleus (blue), and F-actin (green); (scale bar = 100 µm; original magnification ×40). **e** Quantification of infiltrating Ly6G-positive neutrophils; n = 3/gp. Western blot analysis of total and phosphorylated (p) ERK, JNK and p38 in liver after sham or I/R surgery (**f**) or primary mouse HC after H/R (**g**) with treatments as indicated. In vivo: n = 5/gp; 20 ng/mouse MaR1. In vitro: 100 nM MaR1 given as a pretreatment. Images representative of at least 3 independent experiments. Results are expressed as mean ± SEM. *P < 0.05

Previous studies have demonstrated that pro-resolving lipid mediators such as maresins can protect tissues by suppressing damaging inflammation (Sun et al. 2017). Therefore, we examined the serum levels of inflammatory cytokines. Interestingly, we found that MaR1 attenuated systemic inflammation after liver I/R, as shown by significantly lower levels of both serum IL-6 and IL-1β compared to the control mice (Fig. 3a, b), whereas IL-10 levels were not significantly influenced by the treatment of MaR1 (Fig. 3c). Additionally, immunofluorescence staining in liver sections revealed that numbers of LY6G-positive neutrophils were significantly reduced in livers of MaR1-treated mice after I/R insult (Fig. 3d, e). Among numerous signaling programs, the MAPK pathway has been well-recognized in regulating inflammatory response as well as cell survival following I/R injury (Sun et al. 2015). Western blotting revealed that only the expression of p-ERK, but not p-P38 or p-JNK, was suppressed in the MaR1-treated group when compared with the control group after hepatic I/R (Fig. 3f). Similar results were obtained in cultured hepatocytes isolated from WT mice in response to the H/R model with the treatment of MaR1 (Fig. 3g). These results show that MaR1 restrains both systemic and local inflammation in response to hepatic I/R insult in vivo, and the ERK pathway is suppressed by MaR1 in both I/R and H/R stimulation.

### MaR1 suppressed oxidative stress during liver I/R injury

**Fig. 4:**
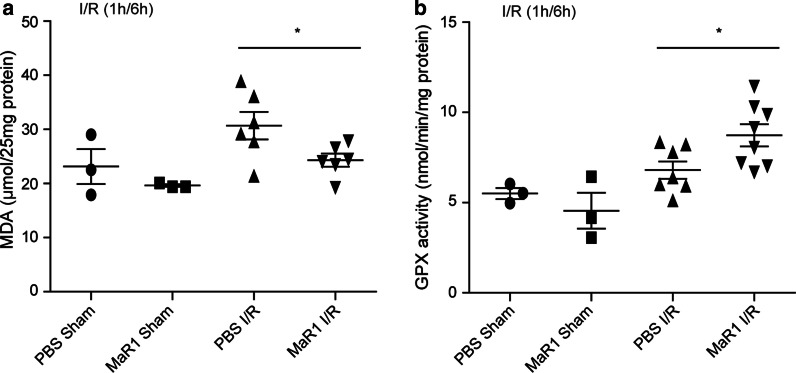
MaR1 suppresses oxidative stress in the liver after I/R injury. Quantitative analysis of concentration of malondialdehyde (MDA) (**a**) and glutathione peroxidase (GPX) (**b**) in liver tissues of WT mice after sham surgery or liver I/R (1 h/6 h) treated with PBS or MaR1 (20 ng/mouse). Results are expressed as mean ± SEM. n = 3 for sham groups; n= 6-8 for I/R group. *P < 0.05

Oxidative stress is also a crucial culprit to hepatocellular damage following I/R injury. To further investigate the effects of MaR1 on oxidative stress induced by liver I/R, the liver levels of malondialdehyde (MDA), a product of lipid peroxidation, (Gu et al. 2018) were evaluated. MDA levels were elevated in the I/R-treated mice compared with those in the sham surgery (Fig. 4a). A reduction in MDA generation was detected in the MaR1-mediated I/R group as compared with the PBS-treated I/R groups (Fig. 4a). Concomitant with the lower MDA levels, a significant increase in the activity of the antioxidant enzyme glutathione peroxidase (GPX) was observed in the livers of the MaR1-treated I/R group as compared with that found in the PBS-treated I/R groups (Fig. 4b). These findings reveal that MaR1 reduces I/R-induced oxidative stress potentially via a mechanism involving enhanced antioxidant enzyme activity (GPX) in the liver.

### Inhibition of lipoxin A4 receptor (ALXR) abolishes the beneficial effects of MaR1 in I/R-induced liver damage

The pro-resolving responses of lipid mediators are transduced by specific G-protein coupled receptors (GPCR) (Chiurchiù et al. 2016). We sought to investigate the implication of SPMs receptors in the effects we observed on MaR1. Since we still have limited information concerning the full spectrum of receptors employed by the different types of SPMs, we mainly focused on the known receptors, ALXR (Gu et al. 2018) and LGR6 (leucine-rich repeat containing G protein–coupled receptor 6) (Chiang et al. 2019), for MaR1. We found that neither mRNA nor protein levels for ALXR and LGR6 were significantly affected after hepatic I/R injury by the presence or absence of MaR1 (Additional file 1: Fig. S2a–c). Similarly, ALXR and LGR6 were unchanged between the normoxia and H/R groups of primary hepatocytes (Additional file 1: Fig. S2d–f). These findings demonstrated that I/R injury had little influence on the expression of ALXR and LGR6.

**Fig. 5:**
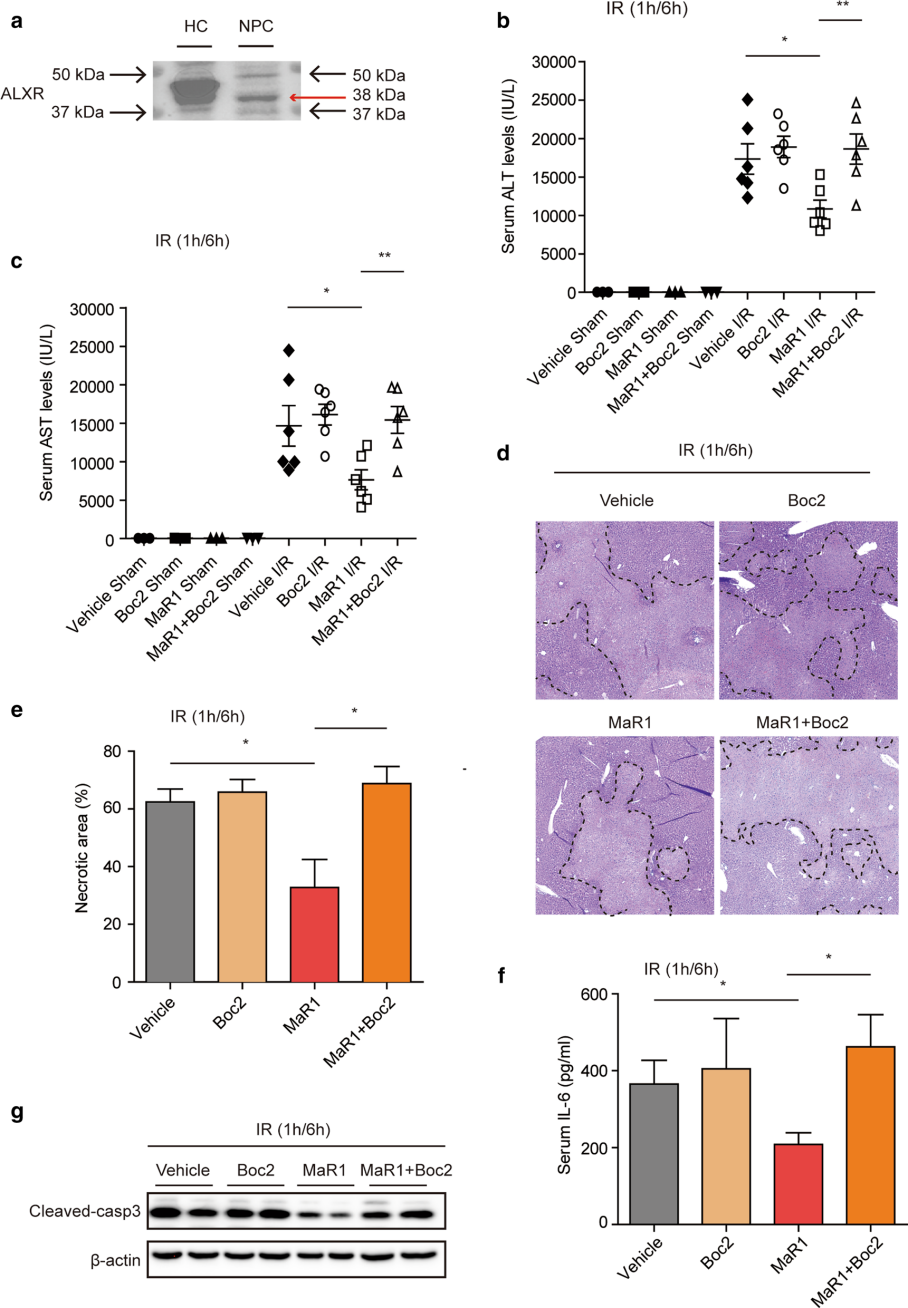
Inhibition of lipoxin A4 receptor (ALXR) abolishes the beneficial effects of MaR1 in I/R-induced liver damage. **a** Western blot of whole-cell lysates from primary mouse HC and NPC assessing protein expression of ALXR at the baseline. WT mice were treated with/without ALXR inhibitor (Boc2; 50 mg/kg, i.p.) 1 h prior to liver I/R with PBS or MaR1 (20 ng/mouse, i.v.) **b**, **c** Serum ALT and AST levels in indicated groups after 6 h reperfusion (n*=*3 in sham groups; n*=*6 in I/R groups). **d** Representative liver H&E staining (original magnification ×20) from indicated groups. **e** Quantitation of liver necrotic areas, quantified in bar graph; n = 5 for each I/R group; **f** Serum IL-6 levels in indicated groups. **g** Western blot analysis of cleaved-caspase 3 in liver tissue from mice subjected to I/R insult in the indicated group. Data are presented as mean ± SEM. *P$<$ 0.05, **P$<$ 0.01

Previous findings demonstrated that MaR1 exerts its function via interacting with lipoxin A4 receptor (ALXR) in CLP-induced sepsis, and the salutary effect of MaR1 could be blocked by Boc2, an ALXR antagonist (Gu et al. 2018). Since ALXR is currently one of the known receptors for MaR1 and an antagonist for ALXR is available, we also explored the role of ALXR on MaR1-mediated protective effects in liver I/R injury. We found that ALXR (38 kDa) was also expressed in NPCs under normoxic conditions (Fig. 5a). Then, the role of ALXR was investigated in vivo. While sALT and sAST levels were significantly lower in MaR1-treated I/R mice, the administration of Boc2 with MaR1 significantly prevented the effect of MaR1 on sALT and sAST levels (Fig. 5b, c). Similarly, the beneficial impact of MaR1 on the morphological changes of liver damage was reversed by Boc2 (Fig. 5d, e). In addition, the MaR1-mediated reduction in serum IL-6 levels and cleaved-casp3 levels induced by MaR1 in I/R injury were also abolished by the pretreatment of Boc2 (Fig. 5f, g). Collectively, these data demonstrate that MaR1 exerts a protective role in I/R-induced liver damage via an ALXR-dependent pathway.

### MaR1-induced Akt activation in hepatic I/R injury is dependent on ALXR

**Fig. 6:**
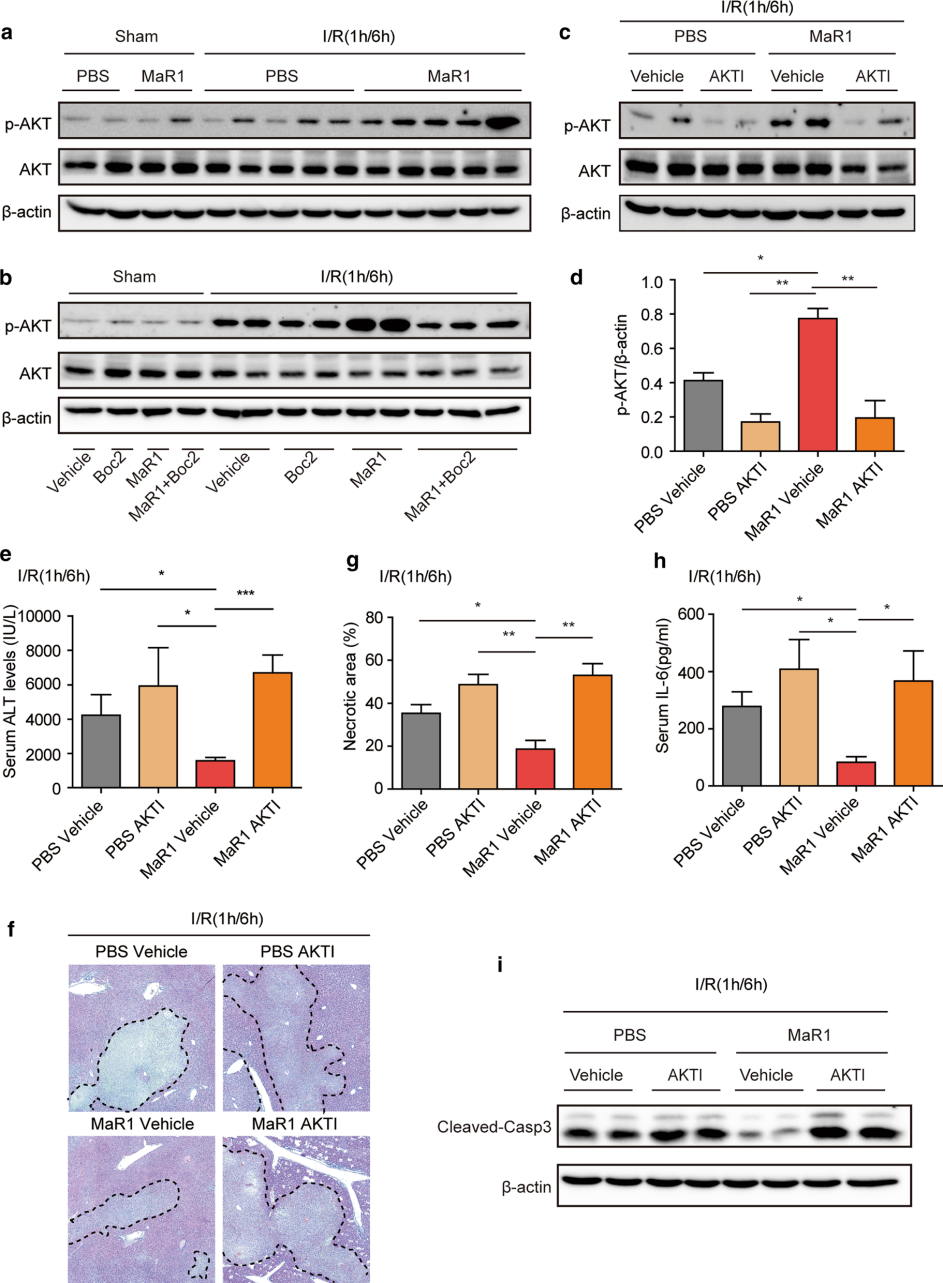
MaR1-induced Akt activation in hepatic I/R injury is dependent on ALXR. **a** Akt and p-Akt protein levels in mice with the treatment of PBS or MaR1 6 h after reperfusion. **b** The expression of Akt and p-Akt was evaluated by Western blot in the liver of the indicated group. **c** Western blot showing the protein levels of total and phosphorylated Akt in mice subjected to vehicle or Akt inhibitor administration with or without the treatment of MaR1. **d** Representative Western blotting and quantitative analyses revealing p-Akt protein expression in the liver of indicated groups. **e** Serum ALT levels were measured in WT mice treated with vehicle or Akt inhibitor in the presence or absence of MaR1and harvested 6 h after I/R (n =5-6 for I/R group). **f** Representative H&E images (original magnification ×20) of liver sections from WT mice treated with vehicle or Akt inhibitor in the presence or absence of MaR1and harvested 6 h post-reperfusion. **g** Percentages of necrotic areas are shown in the indicated I/R groups (n = 3). **h** Serum IL-6 levels of PBS- and MaR1-treated mice subjected to the vehicle or Akt inhibitor and harvested 6 h post-reperfusion as analyzed by ELISA (n = 5-6 for each group). **i** Cleaved-caspase 3 protein expression of PBS- and MaR1-treated mice subjected to the vehicle or Akt inhibitor and liver tissues harvested 6 h after I/R as analyzed by Western blotting. Data are presented as means± SEM. *P$<$0.05, **P$<$ 0.01, ***P$<$0.001

Previous studies showed that Akt signaling is activated following hepatic I/R injury and protects against cell death (Zhang et al. 2014). To further elucidate the regulatory mechanism by which MaR1 affects liver I/R injury, we next investigated the function of the Akt pathway in MaR1-mediated amelioration of liver I/R damage. Consistent with published studies, Akt signaling was activated in I/R-induced liver injury (Fig. 6a). MaR1-treated mice exhibited enhanced Akt activation at 6 h post-reperfusion compared with PBS-treated mice (Fig. 6a), and Boc2 treatment blocked MaR1-mediated activation of Akt (Fig. 6b). To further explore whether the Akt pathway is necessary for MaR1 to protect against hepatic I/R insult, an Akt inhibitor, LY294002, was injected just prior to liver I/R. Western blotting revealed that the Akt inhibitor diminished phosphorylated Akt levels in the liver tissues of PBS-treated and MaR1-treated mice at 6 h after I/R injury when compared with their corresponding control groups (Fig. 6c, d). Measurement of sALT and histological estimation of the liver sections demonstrated that Akt inhibitor treatment significantly accentuated liver damage in both PBS- and MaR1-treated mice (Fig. 6e–g). Most importantly, treatment with Akt inhibitor completely abrogated MaR1-mediated liver protection (Fig. 6e–g). Additionally, Akt inhibition led to a failure of MaR1 to suppress inflammatory responses (IL-6; Fig. 6h) or reduce apoptosis (cleaved-casp3; Fig. 6i). These in vivo results support the conclusion that MaR1 confers protection against liver I/R injury via the activation of an ALXR/Akt signaling pathway.

## Discussion

**Fig. 7:**
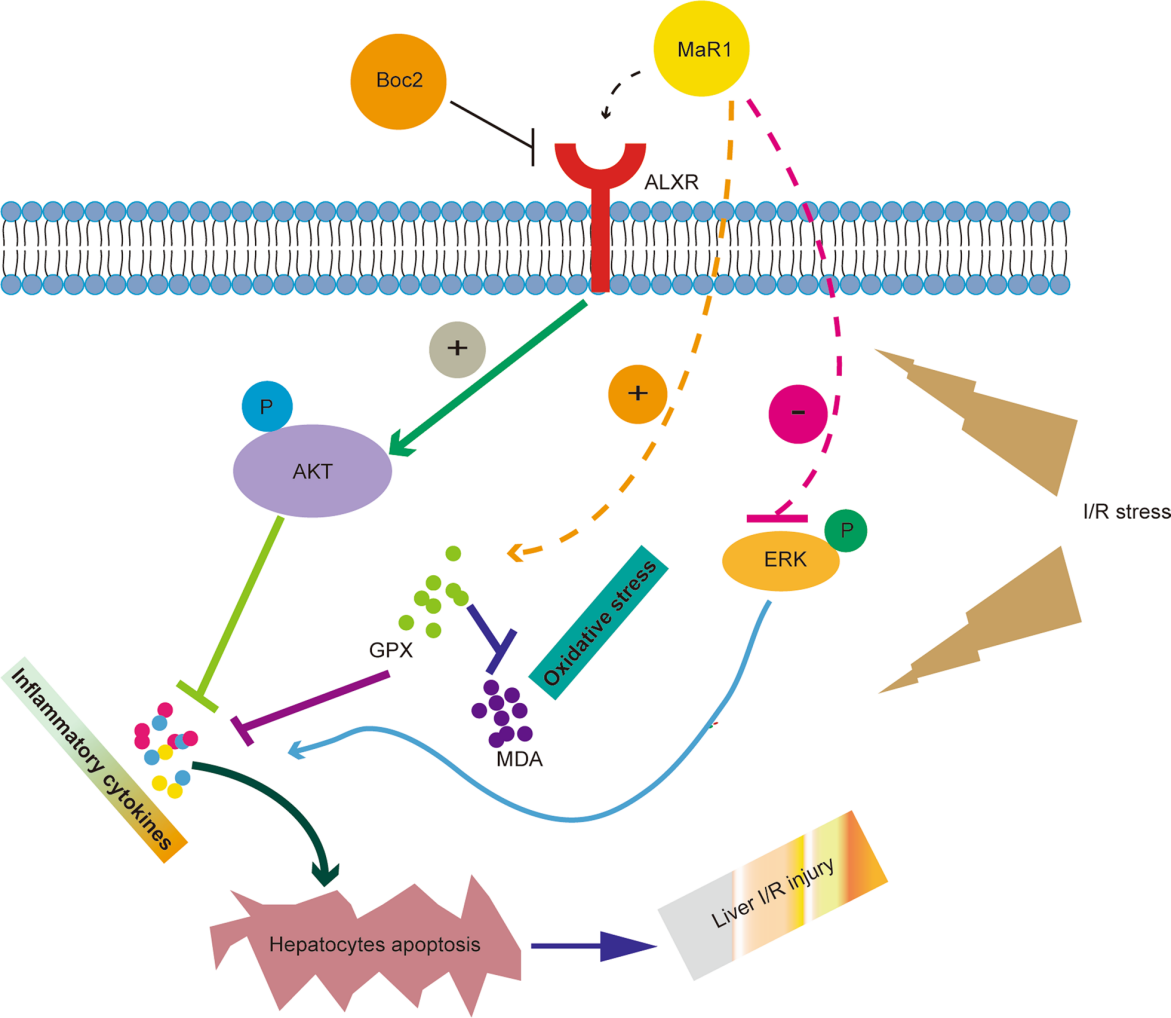
Proposed hepatoprotective signaling mechanisms mediated by MaR1 in liver I/R injury. MaR1 confers protection against I/R-induced liver damage through interacting with ALXR, thus resulting in enhancing downstream p-Akt protein expression. MaR1 also exerts its protective role during hepatic I/R injury by increasing antioxidant enzyme activity (GPX), inhibiting the activation of ERK and suppressing inflammatory responses, and finally contributes to decreased apoptotic cell death

Hepatic I/R can contribute to severe liver damage and is a major clinical problem during liver surgical procedures. Studies of the pathophysiology and underlying mechanisms of liver I/R injury have yielded a number of potential therapeutic alternatives (Gracia-Sancho et al. 2015). However, effective therapeutics in rodent models are limited, and no pharmaceutical therapies specifically target I/R-induced liver injury have been approved for human use. Thus, preventing and attenuating hepatic I/R injury is an unmet clinical need. In this study, we confirm that MaR1 protects against deterioration of liver function in a mouse liver I/R model. The mechanism of hepatoprotection of MaR1 involved anti-inflammatory, anti-apoptosis and anti-oxidative effects during hepatic I/R injury. We show here that MaR1 acts via a known signaling partner, ALXR, to enhance activation of Akt with downstream effects on inflammatory responses and apoptosis resulting in alleviation of I/R-mediated liver damage (Fig. 7).

Previous studies investigating in vivo effects of MaR1 in rats used doses of 4 ng/g body weight given intraperitoneally 1 h prior to liver I/R (Soto et al. 2020). However, we investigated the effects of different doses of MaR1 given at the beginning of reperfusion via the tail vein. We found that a dose of MaR1 of 20 ng/mouse, effective to suppress I/R injury in mice. Thus, the use of MaR1 at the time of I/R could be therapeutically useful.

Numerous studies have provided strong evidence that inflammation-driven by reperfusion-mediated responses is the major contributor to liver damage in I/R injury (Jiménez-Castro et al. 2019, van Golen et al. 2012). Increased levels of circulating inflammatory cytokines are associated with greater liver I/R injury (Liu et al. 2019). Therefore, recent efforts for therapeutic strategies have focused on the direct suppression of inflammation at the reperfusion stage (Datta et al. 2013, Selzner et al. 2003). MaR1 has a series of pro-resolving actions including improved macrophage phagocytosis, diminished neutrophil infiltration and reduced pro-inflammatory cytokine release (Serhan et al. 2009, Buckley et al. 2014). Consistent with these previous investigations, we found that treatment with MaR1 significantly decreased the serum IL-1β and IL-6 levels after hepatic I/R injury. Moreover, there were reduced Ly6G-positive inflammatory cells in MaR1-treated mouse livers. Similarly, we demonstrated that MaR1 negatively regulates ERK signaling, a pivotal component regulating inflammation and cell death, both in vivo and in vitro following liver I/R injury. Taken together, these results demonstrate that MaR1 effectively modulates inflammatory responses after liver I/R. We speculate that this may occur, in part, through the suppression of cellular death.

In addition to inflammation, recent evidence indicated that apoptosis is another essential contributor to hepatic I/R injury (Guo et al. 2020). Apoptosis can also be enhanced by reperfusion-induced inflammatory responses (Jassem et al. 2019). We confirmed that MaR1-treated mice exhibited decreased hepatocyte apoptosis compared with controls after liver I/R. However, this is contradictory to previous findings in a rat liver I/R model, where protein expression of cleaved caspase-3 was dramatically increased with MaR1 treatment (Soto et al. 2020). The reason for the contradictory results is unclear and may be related to species differences, time of treatment with MaR1, or potential adverse/toxic effects of MaR1 in the previously-published study.

In this study, we are the first to demonstrate that the protective role of MaR1 on hepatic I/R injury is independent of NPCs, which suggests that MaR1 has a direct effect on HCs. Hepatic I/R injury is a complicated pathological state, in which oxidative stress also exerts a critical role (Yi et al. 2020). During the reperfusion period, HCs, neutrophils and macrophages can produce ROS, which can trigger the activation of the inflammatory immune responses (Jaeschke 2011). Furthermore, the excess generation of ROS, leading to protein and DNA damage through lipid peroxidation, is regarded as a major cause of oxidative damage to cellular membranes during I/R injury (Rani et al. 2016). In our study, the level of tissue MDA, a main product of lipid peroxidation, was markedly decreased in the MaR1-treated mouse livers following hepatic I/R insult. The eradication of reactive free radicals is dependent on many different antioxidant enzymes, such as superoxide dismutase (SOD), glutathione peroxidase (GPX) and catalase (CAT), which keep the balance between antioxidative effects and oxidative stress responses (Sun et al. 2017). Loss of antioxidant enzymes causes accumulation of free radicals, which further exacerbates I/R-induced injury (Han et al. 2016). Our study reveals a novel effect of MaR1 on the formation of MDA potentially via increased antioxidative enzyme (e.g. GPX) activity during hepatic I/R injury. Thus, it is reasonable to assume that the beneficial effects of MaR1 on liver I/R injury are, at least, partly due to maintenance of the balance between antioxidative and oxidative stresses.

It has been widely accepted that the SPMs, such as MaR1, exert anti-inflammatory actions through direct binding to their corresponding G protein-coupled receptors (GPCRs) (Serhan 2010). However, due to the complicated cellular context, each receptor is capable of interacting with more than one SPM. Previous studies suggested that MaR1 could interact with ALXR (lipoxin A4 receptor) in CLP-induced sepsis in mice to induce protection (Gu et al. 2018). Other studies revealed that resolving E1, another SPM, could induce the generation of endogenous lipoxin A4 in the lung, which was similarly protective (Haworth et al. 2008). In the present study, we confirmed a role for ALXR in mediating the protective effects of MaR1 in liver I/R, although this could be via acting directly at ALXR, or induction of lipoxin A4 production via another MaR1-induced signaling pathway. Further study will be needed to determine which pathway dominates.

Akt is a downstream effector of PI3K signaling shown to modulate multiple cellular effects (Martin et al. 2005). Akt signaling is a known regulator of liver I/R injury (Li et al. 2019), and there is increasing evidence that Akt signaling initiates cell survival through inhibition of apoptosis and improving cell viability (Tsuruta et al. 2002). Our studies showed that MaR1 increases Akt activation/phosphorylation after I/R injury and this was dependent on ALXR signaling. More importantly, Akt upregulation and activation were critical for MaR1-mediated hepatoprotection in liver I/R injury, which is also in line with published studies (Sun et al. 2015, Izuishi et al. 2006).

In conclusion, our present study provides evidence that MaR1 exerts a protective role in I/R-induced injury by reducing the inflammatory response and alleviating hepatocyte apoptosis via ALXR/Akt signaling. These observations broaden our deeper understanding of the direct regulatory role of MaR1 on hepatic I/R insult. Significantly, MaR1 is an endogenous chemical mediator with few identified side effects, suggesting that MaR1 has the potential to be used therapeutically in a wide range of human diseases induced by I/R injury.

## Supplementary Information

**Additional file 1. Figure S1.** In vitro toxicity profile of MaR1. Primary cultured mouse HCs and NPCs were exposed to escalating concentrations of Maresin 1 (MaR1), and cytotoxicity was evaluated using a CKK-8 assay. **Figure S2. **ALXR and LGR6 expression during hepatic I/Rinjury

## Data Availability

The datasets used and/or analyzed during the current study are available from the corresponding author on reasonable request.

## Abbreviations

I/R: Ischemia/reperfusion
H/R: Hypoxia/reoxygenation
MaR1: Maresin 1
ALT: Alanine aminotransferase
AST: Aspartate aminotransferase
IL-1β: Interleukin-1β
IL-6: Interleukin 6
IL-10: Interleukin 10
LDH: Lactate dehydrogenase
MAPK: Mitogen-activated protein kinase
JNK: c-Jun-N-terminal kinase
ERK: Extracellular signal-regulated kinase
ALXR: Lipoxin A4 receptor
LGR6: leucine-rich repeat containing G protein–coupled receptor 6
MDA: Ialondialdehyde
SOD: Superoxide dismutase
GPX: Glutathione peroxidase
CAT: Catalase
HC: Hepatocyte
NPC: Nonparenchymal cell
SPM: Specialized pro-resolving mediator
CLP: Cecal ligation and puncture
Bax: Bcl-2-associated X protein
Bcl-2: B-cell lymphoma 2
LDH: Lactate dehydrogenase
