# Supplementary material for: Maresin 1 protects the liver against ischemia/reperfusion injury via the ALXR/Akt signaling pathway
Source: Mol Med. 2021 Feb 25;27:18. doi: 10.1186/s10020-021-00280-9 (PMC7905895; doi:10.1186/s10020-021-00280-9)

**Supplementary Fig. 1**


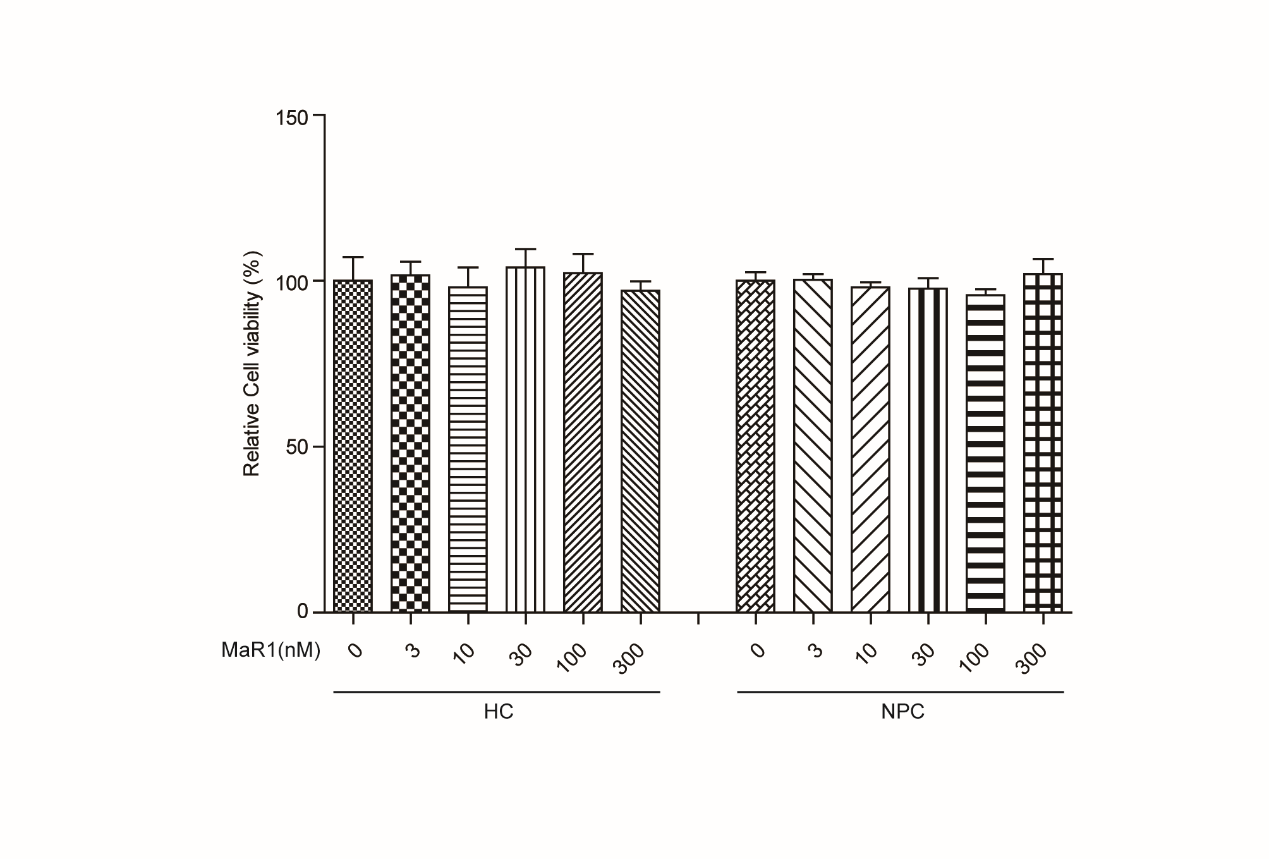


**Supplementary Fig. 1.** In vitro toxicity profile of MaR1. Primary cultured mouse HCs and NPCs were exposed to escalating concentrations of Maresin 1 (MaR1), and cytotoxicity was evaluated using a CKK-8 assay

**Supplementary Fig. 2**

**Supplementary Fig. 2**. ALXR and LGR6 expression during hepatic I/R injury. (A-C) qRT-PCR analysis, immunoblotting of ALXR and LGR6 in WT livers subjected to sham operation and hepatic I/R injury in the indicated group (n=3 for sham group; n = 6 for I/R group). (D-F) ALXR and LGR6 mRNA levels (D) and protein expression (E, F) in primary hepatocytes exposed to normoxia and H/R. All data are shown as mean ± SEM.


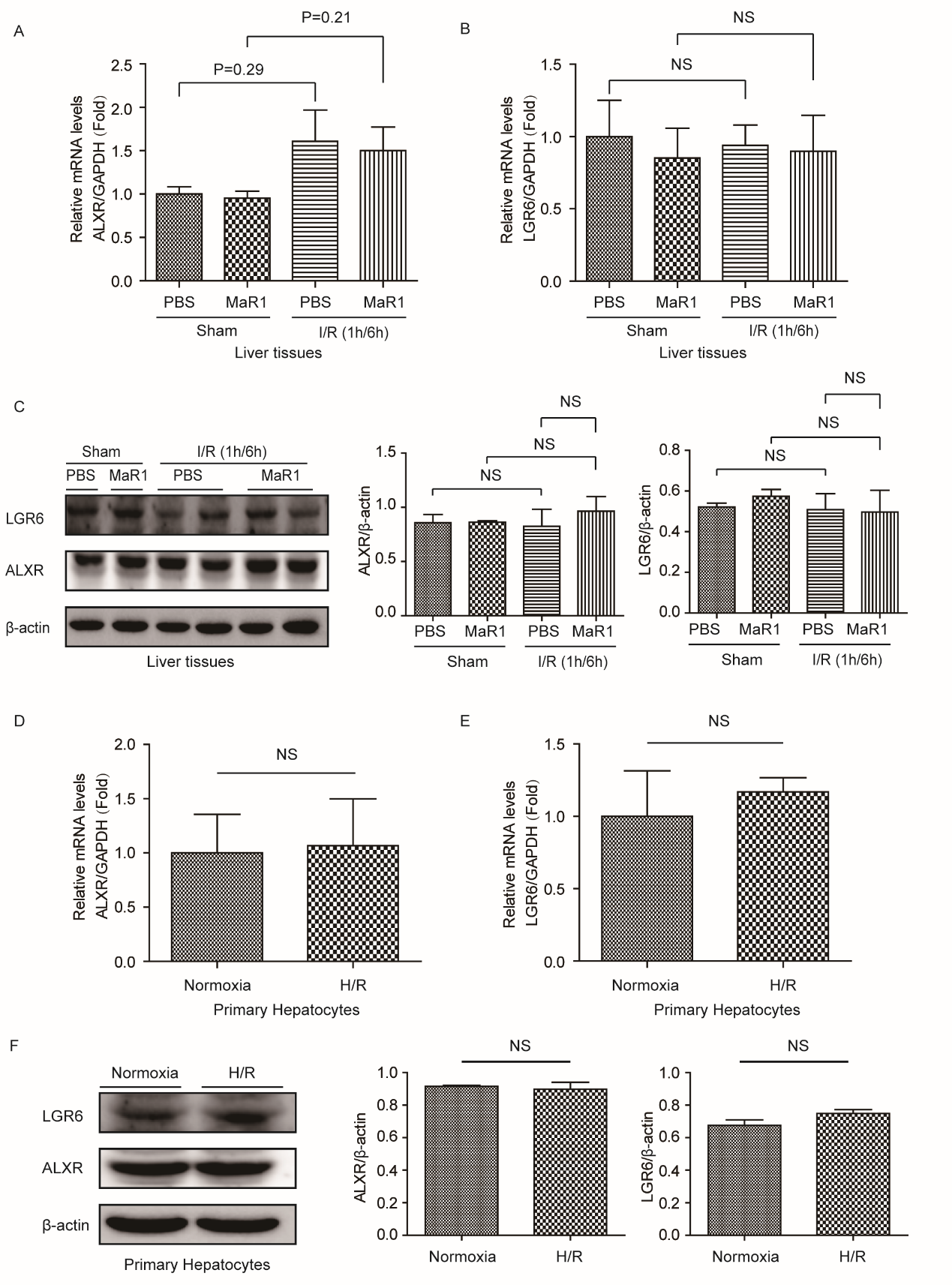

Supplement: Supplementary file 1 — Additional file 1. Figure S1. In vitro toxicity profile of MaR1. Primary cultured mouse HCs and NPCs were exposed to escalating concentrations of Maresin 1 (MaR1), and cytotoxicity was evaluated using a CKK-8 assay. Figure S2. ALXR and LGR6 expression during hepatic I/Rinjury [file 10020_2021_280_MOESM1_ESM.docx]
